# Conserved Regulation of MAP Kinase Expression by PUF RNA-Binding Proteins

**DOI:** 10.1371/journal.pgen.0030233

**Published:** 2007-12-28

**Authors:** Myon-Hee Lee, Brad Hook, Guangjin Pan, Aaron M Kershner, Christopher Merritt, Geraldine Seydoux, James A Thomson, Marvin Wickens, Judith Kimble

**Affiliations:** 1 Howard Hughes Medical Institute, University of Wisconsin-Madison, Madison, Wisconsin, United States of America; 2 Department of Biochemistry, University of Wisconsin-Madison, Madison, Wisconsin, United States of America; 3 Genome Centre of Wisconsin, University of Wisconsin-Madison, Madison, Wisconsin, United States of America; 4 Program in Cellular and Molecular Biology, University of Wisconsin-Madison, Madison, Wisconsin, United States of America; 5 Department of Molecular Biology and Genetics, Johns Hopkins University School of Medicine, Baltimore, Maryland, United States of America; 6 Howard Hughes Medical Institute, Johns Hopkins University School of Medicine, Baltimore, Maryland, United States of America; University of Cambridge, United Kingdom

## Abstract

Mitogen-activated protein kinase (MAPK) and PUF (for *Pu*milio and *F*BF [*fem-3* binding factor]) RNA-binding proteins control many cellular processes critical for animal development and tissue homeostasis. In the present work, we report that PUF proteins act directly on MAPK/ERK-encoding mRNAs to downregulate their expression in both the Caenorhabditis elegans germline and human embryonic stem cells. In *C. elegans,* FBF/PUF binds regulatory elements in the *mpk-1* 3′ untranslated region (3′ UTR) and coprecipitates with *mpk-1* mRNA; moreover, *mpk-1* expression increases dramatically in FBF mutants. In human embryonic stem cells, PUM2/PUF binds 3′UTR elements in both Erk2 and p38α mRNAs, and PUM2 represses reporter constructs carrying either Erk2 or p38α 3′ UTRs. Therefore, the PUF control of MAPK expression is conserved. Its biological function was explored in nematodes, where FBF promotes the self-renewal of germline stem cells, and MPK-1 promotes oocyte maturation and germ cell apoptosis. We found that FBF acts redundantly with LIP-1, the C. elegans homolog of MAPK phosphatase (MKP), to restrict MAPK activity and prevent apoptosis. In mammals, activated MAPK can promote apoptosis of cancer cells and restrict stem cell self-renewal, and MKP is upregulated in cancer cells. We propose that the dual negative regulation of MAPK by both PUF repression and MKP inhibition may be a conserved mechanism that influences both stem cell maintenance and tumor progression.

## Author Summary

The mitogen-activated protein (MAP) kinase (MAPK) enzyme is crucial for regulation of both stem cell maintenance and tumorigenesis. Two conserved controls of MAPK include its activation by RAS signaling and a kinase cascade as well as its inactivation by MAPK phosphatases (MKPs). We identify a third mode of conserved MAPK regulation. We demonstrate that PUF (for *Pu*milio and *F*BF [*fem-3* binding factor]) RNA-binding proteins repress mRNAs encoding MAPK enzymes in both the Caenorhabditis elegans germline and human embryonic stem cells. PUF proteins have emerged as conserved regulators of germline stem cells in *C. elegans, Drosophila,* and probably vertebrates. Their molecular mode of action relies on binding to sequence elements in the 3′ untranslated region of target mRNAs. We report that PUF proteins bind and repress mRNAs encoding C. elegans MPK-1 as well as human ERK2 and p38α. We also report that PUF repression and MKP inactivation function redundantly in the C. elegans germline to restrict MPK-1/MAPK activity and prevent germ cell apoptosis. We suggest that this dual regulation of MAPK activity by PUF and MKP proteins may be a conserved mechanism for the control of growth and differentiation during animal development and tissue homeostasis.

## Introduction

Mitogen-activated protein (MAP) kinases (MAPKs) control many aspects of animal development, including cell proliferation, differentiation, and survival [1]. Most relevant to this work are MPK-1, the primary Caenorhabditis elegans MAPK/ERK homolog [2,3], as well as ERK2 and p38α, two human MAPK homologs [1]. MAPK enzymes are activated by a class of dual specificity kinases that phosphorylate both threonine and tyrosine residues (e.g., [4]) and are inactivated by a class of dual specificity phosphatases, called MAPK phosphatases (MKPs) (e.g., [5,6]). Aberrant ERK2 activation contributes to human developmental disorders, such as Noonan syndrome, Costello syndrome, and cardiofaciocutaneous syndrome (reviewed in [7]); p38α, on the other hand, is thought to inhibit tumor initiation by inducing apoptosis in response to oxidative stress [8]. In mouse embryonic stem cells (mESCs), ERK2 and p38α MAPK signaling promotes differentiation and inhibits self-renewal [9,10].

The C. elegans germline provides a superb model for understanding the molecular controls of stem cells, proliferation, differentiation, and survival [11]. In this simple tissue, germline stem cells are restricted to the distal “mitotic region.” At a molecular level, germline stem cells are maintained by Notch signaling and two RNA-binding proteins, *fem-3* binding factor (FBF)-1 and FBF-2. FBF-1 and FBF-2 (collectively called FBF) are nearly identical and largely redundant proteins that belong to the broadly conserved family of PUF RNA-binding proteins [12,13]. PUF proteins inhibit gene expression by binding regulatory elements in the 3′ untranslated region (3′UTR) of their target mRNAs, thereby controlling their translation or stability [14]. FBF maintains germline stem cells by repressing mRNAs that encode differentiation-promoting regulators. For example, FBF represses *gld-1* and *fog-1* mRNAs*,* which encode regulators that promote entry into meiosis or sperm differentiation, respectively [15,16]. The role of PUF proteins in stem cell maintenance appears to be a conserved and perhaps ancestral function [14,17], but the target mRNAs responsible for this function have not yet been identified. Here, we suggest that MAPK mRNA is one key target.

Once C. elegans germ cells have left the mitotic region, they move proximally and progress through meiosis and gametogenesis. Activated MAPK controls exit from meiotic pachytene and physiological apoptosis during oogenesis [18,19]. Normally, about half of the germ cells progress from pachytene into diakinesis and develop as oocytes, and the other half of the germ cells undergo apoptosis in the proximal pachytene region [19]; however, in mutants with blocked MAPK signaling, germ cells arrest in pachytene and fail to die [18,19]. An antibody that specifically detects activated MAPK, called α-DP-MAPK (for dually phosphorylated MAPK), reveals a dramatic increase in activated MPK-1 just prior to the pachytene to diplotene/diakinesis transition [20,21].

A key inhibitor of C. elegans MPK-1 is LIP-1, a homolog of the dual specificity phosphatase MKP [22]. LIP-1 has two roles in germline development. First, LIP-1 controls the extent of germline proliferation in the mitotic region: wild-type germlines contain significantly more mitotically dividing germ cells than do *lip-1* null mutants [23]. Because the depletion of *mpk-1* rescued the *lip-1* proliferation defect, it seems likely that LIP-1 promotes germline proliferation by inhibiting MPK-1 activity. Second, LIP-1 promotes the G2/M arrest typical of diakinesis, apparently by inhibiting MPK-1 activity after germ cells have exited pachytene [21].

In this paper, we explore the regulatory relationship between PUF proteins and MAPK expression, both in the C. elegans germline and in human embryonic stem cells (hESCs). We find that *mpk-1* mRNA is a direct target of FBF repression in C. elegans and that two human MAPK mRNAs, those encoding ERK2 and p38α, are repressed by human PUM2. We also demonstrate that FBF and LIP-1 function redundantly to inhibit germ cell apoptosis and suggest that this dual regulation of MAPK signaling, which occurs at post-transcriptional and post-translational levels, respectively, may be conserved during diverse cellular processes in animal development and tissue homeostasis.

## Results

### *mpk-1* Expression in the C. elegans Germline

**Figure 1:**
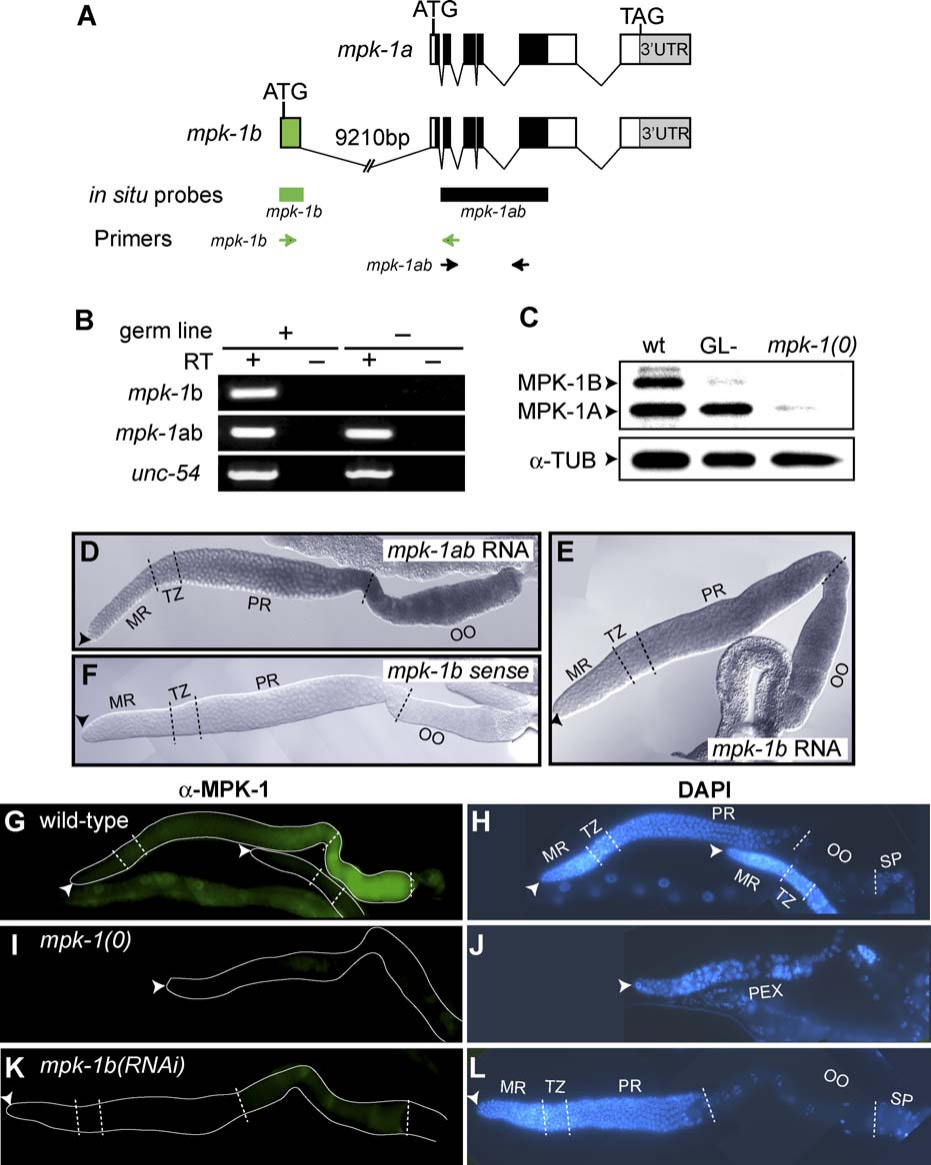
*mpk-1* Expression in the C. elegans Germline (A) Schematics of *mpk-1a* and *mpk-1b* mRNAs. Box, exon; connecting line, intron; ATG, initiation codon; TAG, termination codon. Below schematics: thick bars, extent of probes used for in situ hybridization; arrows, primer pairs used for RT-PCR. (B) Semiquantitative RT-PCR of RNA prepared from adult hermaphrodites that either had an essentially normal germline [*glp-1(q224)* grown at 15 °C], or had virtually no germline [*glp-1(q224)* grown at 25 °C] (see Materials and Methods). *unc-54* was used as a control. (C) Western blot. MPK-1A protein is ≈45 kDa, MPK-1B is ≈55 kDa, and α-TUB is α-tubulin. Proteins were extracted from adult hermaphrodites that were either wild-type (wt), *glp-1(q224)* grown at 25 °C (GL−), or *mpk-1(ga117)* putative null homozygotes *[mpk-1(0)]*. (D–F) In situ analysis of dissected adult hermaphrodite germlines. (D) Total *mpk-1* RNA was assessed using the *mpk-1ab* antisense probe shown in (A). (E) *mpk-1b* RNA was assessed using an isoform-specific antisense probe shown in (A). (F) Negative control, using an *mpk-1b*−specific sense probe. (G–L) Immunocytochemistry of dissected adult hermaphrodite germlines. All were stained using both MPK-1 antibodies (G, I, K) and DAPI (H, J, L). Distal end, arrowhead; dotted lines, boundaries between regions of germline maturation [MR (mitotic region), TZ (transition zone), PR (pachytene region), OO (oocytes), SP (sperm)]; PEX (pachytene exit defect). (G, H) Same wild-type germline. (I, J) Same *mpk-1(0)* germline. (K, L) Same *mpk-1b(RNAi)* germline.

The *mpk-1* gene encodes two major transcripts, *mpk-1a* and *mpk-1b,* which produce MPK-1A and MPK-1B proteins, respectively [2,24] (Figure 1A). To identify which products were expressed in the germline, we performed RT-PCR of RNA prepared from adults that either contained a normal germline (GL+) or contained no germline (GL−). The *mpk-1a* mRNA is contained entirely within *mpk-1b,* but *mpk-1b* harbors a unique exon (Figure 1A). We therefore examined *mpk-1* mRNAs using either *mpk-1ab* primers, which recognize both isoforms, or *mpk-1b*−specific primers. The *mpk-1ab* mRNA was abundant in both GL+ and GL− animals, but *mpk-1b* mRNA was very low or undetectable in GL− animals (Figure 1B). Therefore, *mpk-1b* appears to be enriched in the germline. To corroborate this result, we examined the two MPK-1 proteins in Western blots of protein prepared from wild-type (GL+), GL− mutants, and *mpk-1(ga117)* mutants. MPK-1A was present in both GL+ and GL− animals, but MPK-1B protein was found only in GL+ animals (Figure 1C). We conclude that *mpk-1b* RNA and its MPK-1B protein are predominantly expressed in the germline.

We next investigated the distribution of *mpk-1* mRNA and MPK-1 protein in the germline. After in situ mRNA hybridization of extruded germlines, both *mpk-1ab* and *mpk-1b*−specific probes were low in the mitotic region, increased in the transition zone, and became abundant in the pachytene and oogenic regions (Figure 1D and 1E). No signal was detected with the control *mpk-1* sense probe (Figure 1F). For immunohistochemistry, we used an anti-MAPK/ERK polyclonal antibody that cross-reacts with both MPK-1 isoforms in wild-type animals but is absent from *mpk-1(ga117)* mutants (Figure 1C). The distribution of MPK-1 protein was similar to that of *mpk-1* mRNA: MPK-1 protein was low in the distal germline (e.g., mitotic region, transition zone), was increased in the proximal pachytene region, and became abundant in developing oocytes (Figure 1G and 1H). Essentially no signal was seen in *mpk-1(ga117)* mutant germlines (Figure 1I and 1J). To investigate the isoform expressed, we depleted *mpk-1b* mRNA by RNA interference (RNAi); the specific elimination of MPK-1B was verified by Western blots (unpublished data). MPK-1 protein was essentially absent from *mpk-1b* RNAi germlines, except for a low signal in developing oocytes (Figure 1K and 1L). We conclude that MPK-1B is the predominant MPK-1 isoform in the germline.

### FBF Represses *mpk-1b* Expression in the Distal Germline

**Figure 2:**
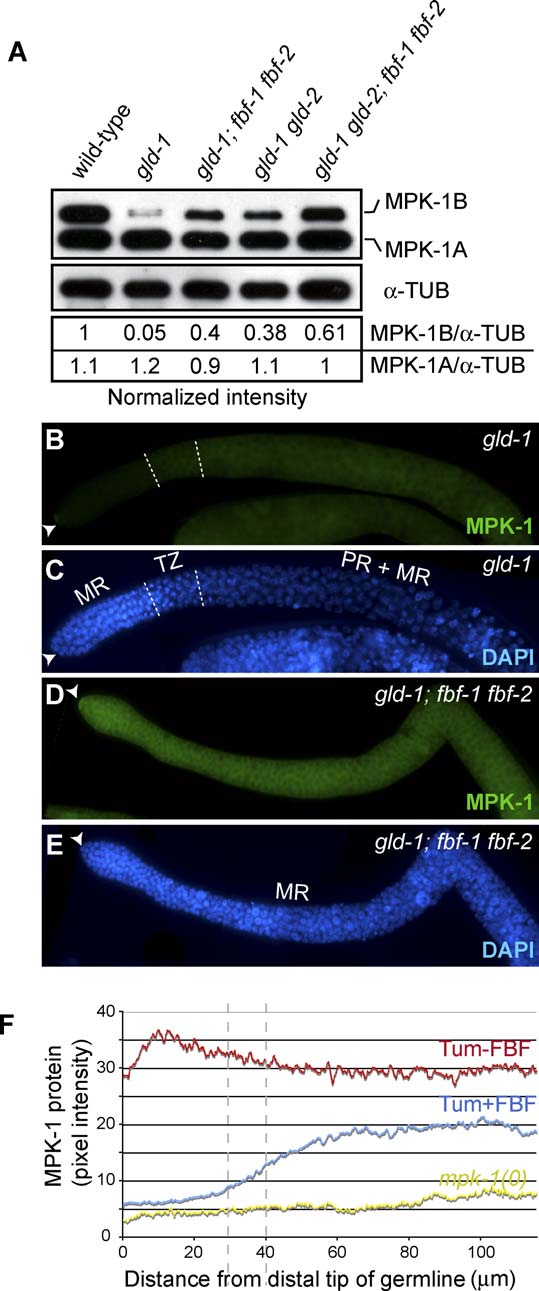
FBF Represses *mpk-1b* Germline Expression (A) Western blot analysis. MPK-1B abundance increases when FBF is removed. Loading control, α-tubulin. Band intensity was measured using ImageJ software. (B–E) Dissected adult hermaphrodite germlines with FBF (B, C) and without FBF (D, E); genotypes noted in images. Distal end, arrowhead; dotted lines, boundaries between regions of germline maturation (same conventions as detailed in Figure 1G–1L legend). (B) MPK-1 staining in tumorous germlines that contain FBF. (C) DAPI staining of germline shown in (B). (D) MPK-1 staining in tumorous germlines that have no FBF. (E) DAPI staining of germline shown in (D). Germlines were treated identically, and images were taken with the same settings at the same magnification for comparison. (F) Quantitation of MPK-1 protein in Tum+FBF (*gld-1*, *n* = 7), Tum−FBF (*gld-1; fbf-1 fbf-2*, *n* = 5), and *mpk-1(0)* (*n* = 3) mutants. The intensity of MPK-1 protein was quantified using ImageJ software. The *x*-axis represents distance from distal tip of the germline, and the *y*-axis is pixel intensity. Dotted lines show boundaries of transition zone in Tum+FBF (*gld-1*) mutants; Tum−FBF (*gld-1; fbf-1 fbf-2)* mutants do not have a transition zone.

To determine if FBF might repress *mpk-1* expression, we compared the abundance of MPK-1 protein in germlines that either had wild-type FBF (both *fbf-1* and *fbf-2*) or no FBF (neither *fbf-1* nor *fbf-2*). For this study, we could not examine a simple *fbf-1 fbf-2* double mutant, because that animal does not maintain mitotically dividing germ cells [15]. Instead, we examined *mpk-1* expression in tumorous (Tum) germlines that have robust germ cell proliferation both with and without FBF. In *gld-1* mutants (Tum+FBF), MPK-1B was about 8-fold lower than in *gld-1; fbf-1 fbf-2* mutants (Tum−FBF) (Figure 2A, lanes 2 and 3). MPK-1B was also lower in *gld-1 gld-2* mutants (Tum+FBF) than in *gld-1 gld-2; fbf-1 fbf-2* mutants (Tum−FBF) (Figure 2A). By contrast, MPK-1A levels were equivalent in these strains (Figure 2A). Therefore, FBF affects MPK-1B, but not MPK-1A, abundance.

To visualize where within the germline FBF affects MPK-1 expression, we stained dissected germlines with both the MPK-1 polyclonal antibody and DAPI, and we quantitated levels with ImageJ software. Consistent with the Western blot data, MPK-1 was lower in *gld-1* germlines than in *gld-1; fbf-1 fbf-2* germlines (Figure 2B–2F). This difference was particularly striking within the mitotic region, where MPK-1 was about 5-fold lower in Tum+FBF than in Tum−FBF germlines (Figure 2B–2F). We also stained *fbf-1* single mutant germlines, which maintain a mitotic region but are compromised for full FBF activity; in about 20% of dissected germlines, MPK-1 protein was detected in the distal mitotic region (unpublished data). We conclude that FBF maintains a low level of MPK-1 protein in the distal germline.

### FBF Binds FBF Binding Elements in *mpk-1* 3′UTR

**Figure 3:**
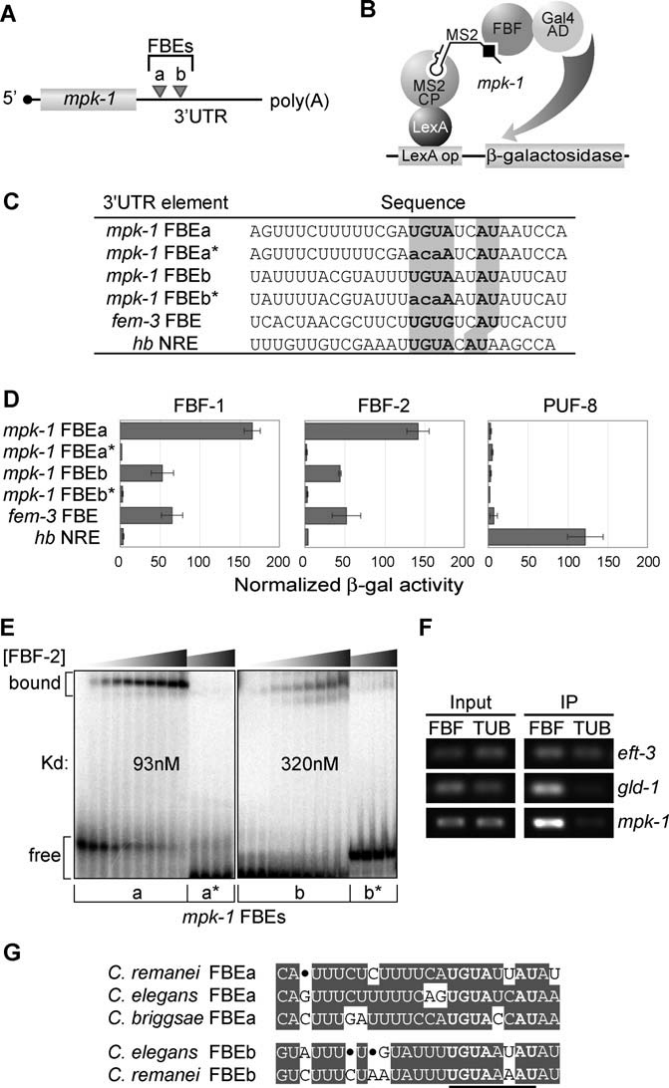
FBF Binds Specifically to FBEs in *mpk-1* 3′UTR (A) Two predicted FBF binding elements in *mpk-1* 3′UTR. (B) Schematic of yeast three-hybrid assay. Briefly, a hybrid RNA carrying the query sequence can bridge the LexA-MS2 and GAL4AD-FBF hybrid proteins if FBF binds, but it cannot bridge them if FBF fails to bind. (C) Nucleotide sequences of predicted FBEs, aligned in register with their conserved UGURHHAU motifs (bold in gray boxes). Each wild-type sequence is followed by its mutant (*), in which UGU is replaced by aca (mutated nucleotides are lowercase). Controls included the *fem*-3 FBE in the *fem-3* 3′UTR, previously called the PME [12], which served as a positive control for FBF binding, and the *hb (hunchback)* NRE, which served as a negative control for FBF binding and a positive control for PUF-8 binding [55]. (D) Three-hybrid interactions assayed by β-galactosidase activity. Nomenclature and conventions are the same as in (C). Standard deviation bars were calculated from three independent experiments. (E) Purified FBF-2 binds *mpk-1* FBEa and *mpk-1* FBEb in gel mobility assays, but not to mutants (*) with an altered consensus as detailed in (C). Apparent affinities of MPK-1 FBEa and FBEb are 93 nM and 320 nM, respectively. (F) Coimmunoprecipitation of *mpk-1* mRNA with an epitope-tagged FBF. *eft-3* served as a negative control, and *gld-1* served as a positive control [15]. (G) Sequence alignment of *mpk-1* FBEs from C. elegans, *C. briggsae,* and C. remanei.

 FBF binds specifically to FBFbinding elements (FBEs) within the 3′UTR of its direct target mRNAs [12,13,15,16,23,25]; potential FBEs can be recognized by a consensus sequence (UGURHHAUW) [“R,” purine; “H,” not G; “W,” A or U] [26]. The *mpk-1* 3′UTR possesses two potential FBEs that conform to this sequence (Figure 3A). To assess FBF binding to these predicted *mpk-1* FBEs, we used both yeast three-hybrid (Figure 3B and 3D) and gel retardation assays (Figure 3E). Yeast three-hybrid interactions were monitored by production of β-galactosidase from a lacZ reporter (Figure 3D). The *mpk-1* FBEa and FBEb interacted with both FBF-1 and FBF-2 in three-hybrid assays (Figure 3C and 3D) and bound to purified recombinant FBF-2 in gel shift assays (Figure 3E). Furthermore, those interactions were specific: wild-type *mpk-1* FBEa and FBEb bound FBF, but not PUF-8 (Figure 3D) or PUF-5 (unpublished data), and that binding was disrupted by mutations of the UGU in the consensus binding site (Figure 3C–3E, FBE* mutant changed UGU to aca). The apparent *K_d_* values for *mpk-1* FBEa and FBEb were about 93 nM and 320 nM, respectively. We conclude that the *mpk-1* 3′UTR bears two FBEs and that FBEa appears to have higher affinity for FBF than does FBEb.

We next asked whether FBF protein associates with *mpk-1* mRNA in the nematode. Specifically, we prepared C. elegans extracts from animals carrying either a rescuing epitope-tagged GFP::FBF or a control GFP::tubulin (TUB), and incubated those extracts with immobilized GFP antibodies to immunoprecipitate (IP) associated mRNAs. We then used RT-PCR to assess either *mpk-1* or control mRNAs (*eft-3*, negative control; *gld-1*, positive control). *mpk-1* mRNA was reproducibly enriched in the IP from GFP::FBF-bearing animals compared to that from the GFP::TUB animals (Figure 3F). Therefore, FBF is likely to bind directly to the *mpk-1* mRNA in vivo. Interestingly, the *mpk-1* FBEa is conserved in three *Caenorhabditis* species: *C. elegans, C. briggsae,* and *C. remanei* (Figure 3G). We conclude that the *mpk-1* 3′UTR possesses FBEs and that FBF repression of *mpk-1* expression is direct.

### FBF and LIP-1 Function Redundantly to Control Distribution of Activated MPK-1

**Figure 4:**
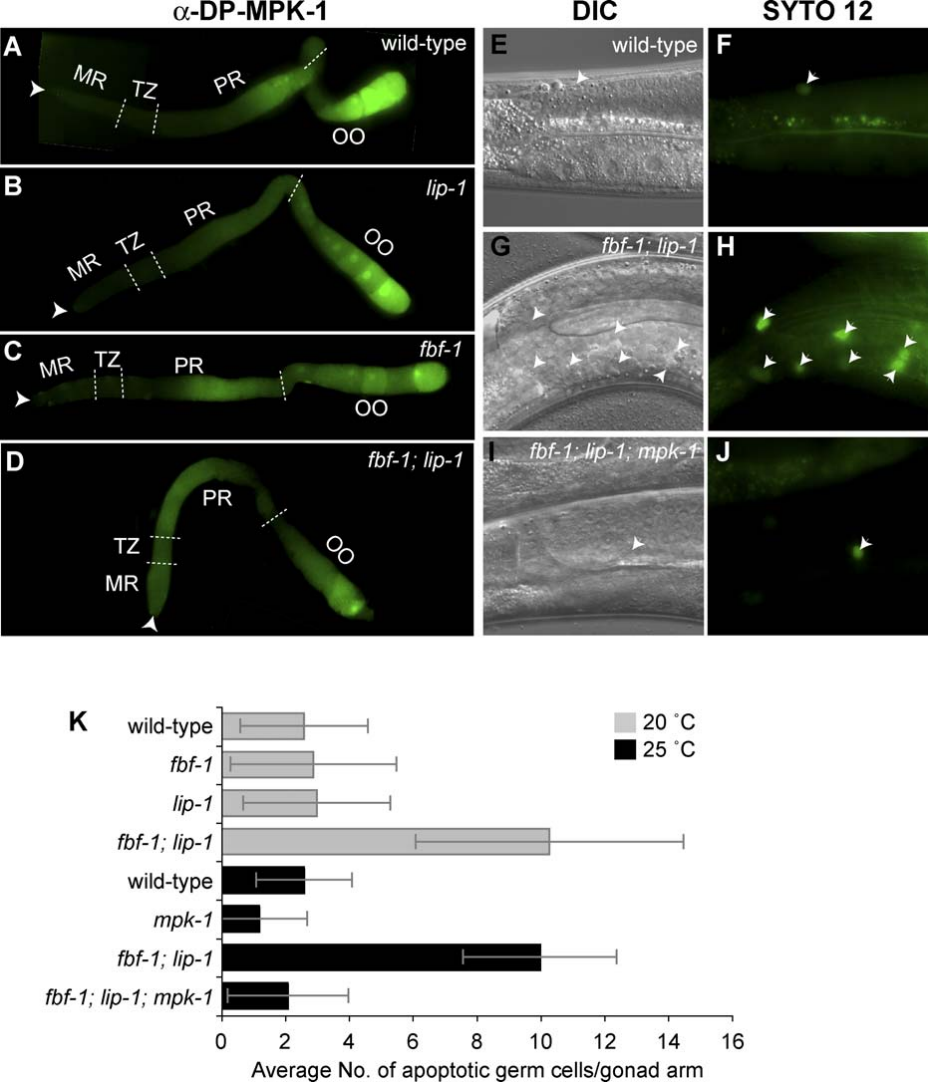
FBF and LIP-1 Function Redundantly to Control Distribution of Activated MPK-1 and to Protect Germ Cells From Apoptosis (A–D) Dissected adult germlines stained with α-DP-MAPK antibody, which is specific for activated MPK-1. Germlines were treated identically, and images were taken with the same settings at the same magnification. Conventions are same as described in Figure 1. (E–J) Nomarski (left column) and SYTO 12 staining (right column) of adult hermaphrodite germlines (24 h after L4 at 20 °C or 18 h past L4 at 25 °C). The vital dye SYTO 12 stains apoptotic germ cells (arrowheads in F, H, and J). (K) Average number of apoptotic germ cells per gonad arm. Standard deviation bars were calculated from three independent experiments.

The C. elegans homolog of MAPK phosphatase, LIP-1, behaves genetically as an inhibitor of MAPK activity and is likely to inactivate MPK-1 in germ cells (see Introduction) [21,23]. Therefore, MAPK is negatively regulated in the germline by two distinct mechanisms: FBF represses *mpk-1* expression (present work) and LIP-1 inhibits MAPK activity. To test the possibility that FBF and LIP-1 might function redundantly to control the distribution of activated MPK-1, we used the α-DP-MAPK monoclonal antibody, which recognizes the active form of MAPK by its dual phosphorylation (DP). In wild-type germlines, activated MPK-1 was not detected in the distal germline (e.g., mitotic region, transition zone) but became abundant in the proximal part of the pachytene region and in maturing oocytes (Figure 4A) [20,21]. A similar distribution was seen in *fbf-1* and *lip-1* single mutants (Figure 4A–4C). By contrast, activated MPK-1 was broadly distributed in *fbf-1; lip-1* double mutant germlines, extending all the way to the distal tip (Figure 4D). We conclude that activated MPK-1 is subject to two redundant modes of downregulation: FBF acts post-transcriptionally to repress *mpk-1* mRNA and LIP-1 is likely to act post-translationally to inhibit MPK-1 activity.

### FBF and LIP-1 Function Redundantly to Protect Germ Cells from Apoptosis

In wild-type C. elegans hermaphrodites, physiological germ cell apoptosis requires MPK-1 activation [19]. Strong loss-of-function mutations in any of the genes of the RAS/MPK-1 pathway interrupt germ cell apoptosis [19], but germ cell apoptosis does not increase in *let-60/Ras* gain-of-function *(gf)* mutants [19,27]. Although MPK-1 activity is much higher in *let-60(gf)* germlines than in wild-type, the distribution of activated MPK-1 is similar in *let-60(gf)* and wild-type germlines [21]. We hypothesized that germ cell apoptosis might be regulated by the distribution of activated MPK-1 rather than its quantity at the site of apoptosis. To test this idea, we counted the number of germ cell deaths in adult hermaphrodite germlines, using the vital dye SYTO 12 (Molecular Probes) to detect apoptotic corpses. In these experiments, we used *fbf-1* mutants to deplete but not eliminate FBF activity: FBF-2 provides sufficient FBF to maintain germline stem cells. Wild-type hermaphrodite germlines had about 2.6 germ cell corpses per gonad arm (Figure 4E, 4F, and 4K), and a similar number was seen in *fbf-1* and *lip-1* single mutants (Figure 4K). However, in *fbf-1; lip-1* double mutants, the number of germ cell corpses increased dramatically (Figure 4G, 4H, and 4K).

To determine if the increased germ cell apoptosis in *fbf-1; lip-1* mutants depends on MPK-1 activity, we used *mpk-1(ga111),* a temperature-sensitive mutation. Specifically, we compared the number of germ cell corpses after shifting *fbf-1; lip-1* double mutants and *fbf-1; lip-1; mpk-1(ga111ts)* triple mutants to restrictive temperature (25 °C). Whereas the *fbf-1; lip-1* double mutant displayed excess germ cell death, the *fbf-1; lip-1; mpk-1(ga111ts)* triple mutant had far fewer corpses (Figure 4I, 4J, and 4K). Therefore, MPK-1 activity is required for the increased apoptosis in *fbf-1; lip-1* double mutants. We conclude that FBF and LIP-1 proteins act redundantly to inhibit MPK-1 activity and promote germ cell survival.

### PUF Binding to MAPK 3′UTRs Is Conserved in Humans

**Figure 5:**
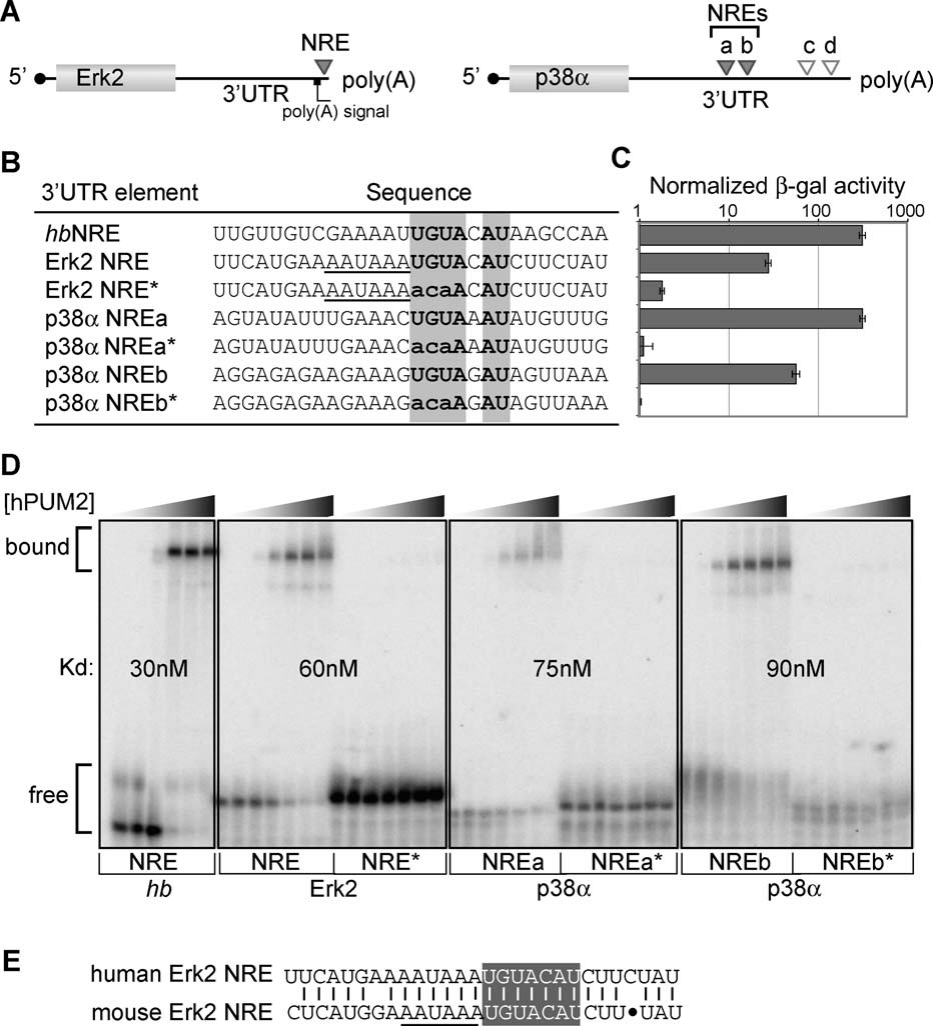
Conservation of PUF Binding to Regulatory Elements in Human Erk2 and p38α 3′UTRs (A) Putative PUM2 binding elements (NREs) in Erk2 and p38α 3′UTRs; filled triangles, elements that bound in vitro; empty triangles, elements that did not bind in vitro. (B) Nucleotide sequence of predicted NREs. Sequences are aligned in register with their conserved UGUANAU motif (bold in gray boxes). Mutated nucleotides are lowercase. (C) Three-hybrid interactions assayed by β-galactosidase activity. Standard deviation bars were calculated from three independent experiments. (D) Purified PUM2 binds Erk2 NRE as well as p38α NREa and NREb in gel mobility assays, but does not bind mutants (*) with an altered consensus as detailed in (B). (E) Sequence alignment of Erk2 NREs from human and mouse.

We next investigated the possibility that PUF RNA-binding proteins might also control MAPK expression in humans. This idea was inspired in part by the knowledge that the human PUF protein, PUM2, is expressed abundantly in both human embryonic stem cells (hESCs) and human germline stem cells [28] and in part by the conserved PUF role in stem cell maintenance (see Introduction). Predicted Pumilio binding elements (known as NREs [nanos response elements]) were sought using UGUANAU as a core consensus [29]. The Erk2 3′UTR possesses a putative NRE immediately adjacent to the cleavage and polyadenylation hexanucleotide sequence (AAUAAA) (Figure 5A and 5B), and the p38α 3′UTR has four putative NREs (Figure 5A). In yeast three-hybrid assays, the Erk2 NRE, p38α NREa, and p38α NREb all interacted specifically with PUM2 (Figure 5C). Moreover, the wild-type NREs in Erk2 and p38α bound purified PUM2 protein in gel shift assays (Figure 5D), but mutant NREs (NRE*) with an altered consensus (Figure 5B) did not (Figure 5D). We next asked whether an Erk2 NRE is conserved in mouse Erk2 3′UTR. Intriguingly, the Erk2 NRE is conserved in human and mouse 3′UTRs, both being located next to the hexanucleotide sequence (Figure 5E). We conclude that PUM2 protein binds to Erk2 and p38α 3′UTRs and that PUF binding to MAPK 3′UTRs is highly conserved.

### PUM2 Binding Elements in Erk2 and p38α 3′UTRs Confer Repression in hESCs

**Figure 6:**
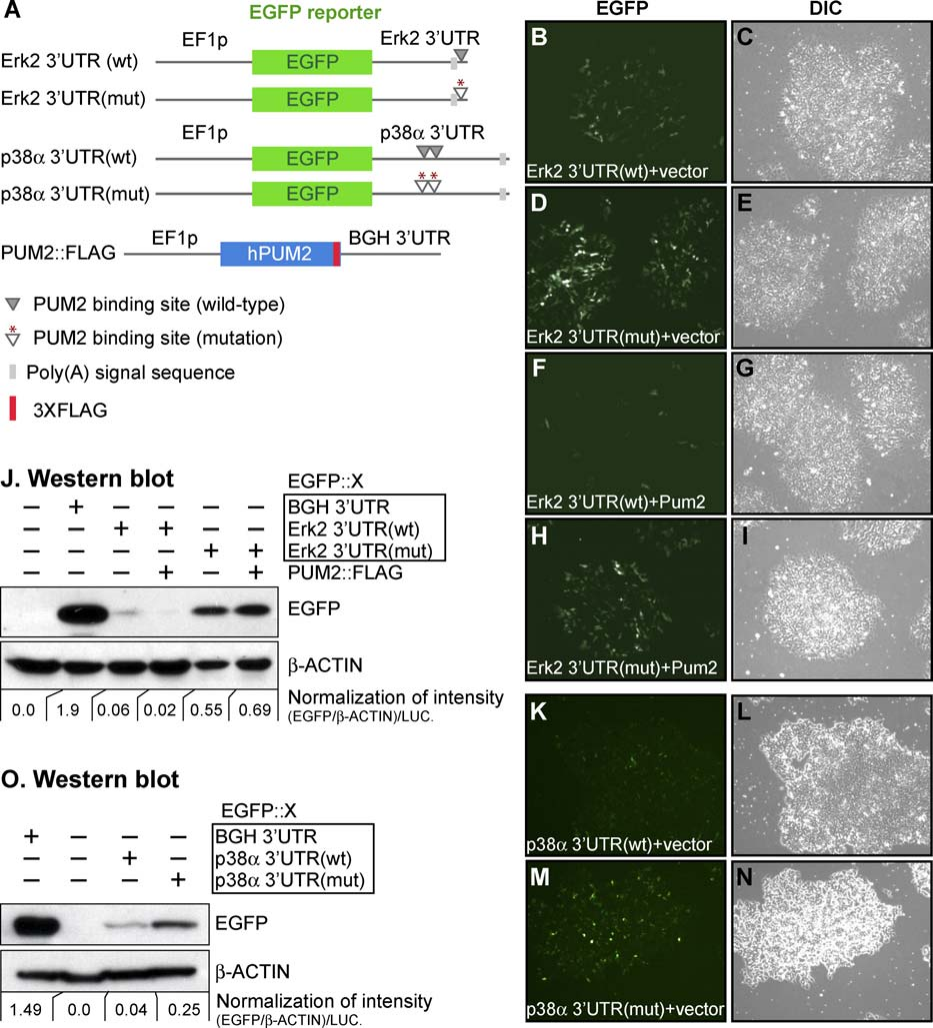
The Erk2 and p38α 3′UTRs Control Reporter Expression in hESC (A) Schematic diagram of DNA constructs used for reporter assays. EGFP reporters all use the same EF1p promoter and contain one of the following 3′UTRs: Erk2 or p38α wild-type (wt) or Erk2 or p38α mutant (mut). The Pum2::FLAG construct contained PUM2 protein coding region plus three copies of FLAG sequences. All transfections included a luciferase-expressing plasmid to control for transfection efficiency (see Materials and Methods). (B–I, K–N) Photomicrographs of hESCs; experimental plasmids are noted in images. All steps were performed identically to compare EGFP expression levels; images shown were representative of at least three independent experiments. (J, O) Western blot analysis. PUM2 represses expression of EGFP reporter constructs bearing either Erk2 or p38α 3′UTRs. The intensities were normalized using β-actin to control for cell number and luciferase (LUC) to control for transfection efficiency.

To test if PUM2 controls Erk2 and p38α expression, we performed a series of enhanced green fluorescent protein (EGFP)-based reporter assays in hESCs. Specifically, we fused an EGFP reporter to the Erk2 3′UTR that contained either a wild-type NRE, Erk2 3′UTR(wt), or a mutated NRE, Erk2 3′UTR(mut) (Figure 6A). We transfected these constructs along with a transfection control into hESCs and monitored EGFP expression 24 h later. We first observed EGFP using fluorescence microscopy and then determined expression levels by Western blot analysis (Figure 6B–6J). As a control, we used a reporter carrying a 3′UTR without any predicted NREs (EGFP::BGH [bovine growth hormone] 3′UTR). hESCs carrying the EGFP::BGH 3′UTR reporter expressed EGFP at a very high level (Figure 6J). By contrast, hESCs transfected with the Erk2 3′UTR(wt) reporter had much less EGFP (Figure 6B, 6C, and 6J). To ask if the NRE is critical for this low expression, we assayed Erk2 3′UTR(mut), a reporter with three altered nucleotides in the NRE consensus (UGU to aca) (Figure 6A). This Erk2 3′UTR(mut) reporter produced about 9-fold more EGFP than the Erk2 3′UTR(wt) reporter (Figure 6D, 6E, and 6J). We speculated that endogenous PUM2 might repress expression of the Erk2 3′UTR(wt) reporter but not the Erk2 3′UTR(mut) reporter. Attempts to use siRNA to silence endogenous PUM2 were not successful. We therefore cotransfected hESCs with the EGFP reporters and PUM2::FLAG (Figure 6A), and we found that PUM2::FLAG dramatically repressed Erk2 3′UTR(wt) expression (Figure 6F, 6G, and 6J) but did not repress Erk2 3′UTR(mut) expression (Figure 6H, 6I, and 6J).

We next asked if reporters carrying the p38α 3′UTR were also controlled in an NRE-dependent manner. To this end, we transfected hESCs with either of two EGFP-based reporter genes, p38α 3′UTR(wt) or p38α 3′UTR(mut) (Figure 6A). As found for the Erk2 reporter, the wild-type, but not the mutant, p38α 3′UTR was capable of efficiently repressing expression from the EGFP reporter gene in hESCs (Figure 6K–6O). In this case, expression from p38α 3′UTR(wt) was about 6-fold lower than that from p38α 3′UTR(mut) (Figure 6O). Taken together, we conclude that the PUM2 binding elements present in both Erk2 and p38α 3′UTRs mediate repression in hESCs.

## Discussion

**Figure 7:**
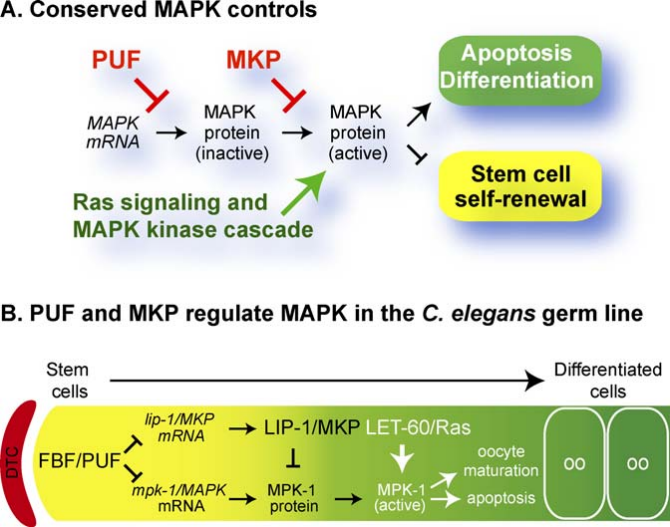
Regulation of MAPK Activity by PUF and MKPs (A) Conserved positive and negative regulators of MAPK expression and activity. See text for further explanation. (B) MAPK regulation in the C. elegans germline. The distal end of the germline is controlled by Notch signaling from the distal tip cell (DTC), which provides the stem cell niche [11]. FBF/PUF RNA-binding proteins are present in the distalmost germ cells, which include stem cells. FBF maintains germ cells in a naïve and undifferentiated state, in part by repression of *mpk-1* expression (present work). In addition, FBF represses *lip-1/MKP* mRNA in the stem cell region [23]; more proximally, where FBF abundance decreases, LIP-1/MKP inhibits MPK-1/MAPK activity; yet more proximally, LET-60/RAS activates MPK-1/MAPK to promote oocyte differentiation and apoptosis. OO, oocyte.

The MAPK enzyme is controlled by several conserved pathways (Figure 7A). Best understood is its activation by RAS and a kinase cascade, a pathway that has been conserved in virtually all eukaryotic cells [4]. In addition, MAPK is inhibited by the conserved dual specificity MKPs [5,6]. Here, we show that the PUF RNA-binding proteins are another broadly conserved mechanism of MAPK control. We demonstrate that PUF proteins control the expression of MAPK-encoding mRNAs in both the C. elegans germline and hESCs. We also show that PUF repression and MKP inhibition are redundant in their ability to restrict activated MAPK and prevent apoptosis in the C. elegans germline. We propose that the dual regulation of MAPK signaling by PUF repression and MKP inhibition may be a conserved means of influencing both stem cells and tumor progression.

### Conserved PUF Repression of MAPK-Encoding mRNAs

Both PUF RNA-binding proteins and MAPK enzymes are highly conserved from yeast to humans. In this paper, we show that PUF proteins directly bind to 3′UTR regulatory elements in MAPK-encoding mRNAs and thereby control the generation of MAPK protein. Specifically, C. elegans FBF binds and regulates *mpk-1* expression in germ cells, and human PUM2 binds and regulates Erk2 and p38α 3′UTRs in hESCs. Similarly in yeast, the Mpt5 PUF protein inhibits Ste7/MAPKK expression to regulate the filamentation-specific MAPK pathway [30]. Therefore, an ancient relationship appears to exist between the PUF RNA-binding proteins and MAPK signaling. To our knowledge, our work provides the only direct link between PUF proteins and MAPK-encoding mRNAs. Because this direct connection exists in both C. elegans and humans, we suggest that it may represent a broadly conserved regulatory relationship among metazoans.

Figure 7A places PUF repression into a conserved pathway of MAPK control. Specifically, PUF proteins are responsible for the post-transcriptional repression of MAPK mRNAs; mechanistically, this could be achieved by controlling either their translation or stability. PUF proteins were originally thought to control mRNA stability in yeast but to control mRNA translation in animals [14], but as more examples of PUF-controlled mRNAs have surfaced, it has become clear that this generalization is too simple. For example, C. elegans FBF controls the stability of *lip-1* mRNA [23]*,* and yeast Mpt5 controls the translation of HO mRNA [31]. Regardless of mechanism, our work shows conclusively that PUF proteins are direct regulators of MAPK-encoding mRNAs.

### Dual Negative Regulation of MAPK and Its Effect on Apoptosis

MAPK is a key regulator of programmed cell death, among its other roles during animal development [1]. In this work, we investigated the function of PUF repression and MKP inhibition in the control of apoptosis in the C. elegans oogenic germline. In wild-type animals, about half of the germ cells die and the other half begin oocyte maturation (Figure 7B) [19]. Indeed, activated MPK-1 is most abundant where germ cells either die or begin oogenesis [20,21]. In mutants lacking either FBF-1/PUF or LIP-1/MKP, the distribution of activated MPK-1 is essentially normal and the number of germ cells that undergo cell death is also normal. By contrast, in double mutants lacking both FBF-1 and LIP-1, activated MPK-1 extends all the way to the distal end of the germline, where it is normally never seen, and apoptosis increases dramatically. This result suggests two things. First, because distribution of activated MAPK affects number of apoptotic germ cells, the decision to die may be programmed at a location distal to their actual site of death. Second, and perhaps most important for this work, the distribution of activated MAPK and the number of germ cell deaths are controlled redundantly by FBF repression and LIP-1 inhibition.

MAPK inhibition is ensured in the C. elegans germline, at least in part because FBF represses *lip-1* mRNA in addition to its control of *mpk-1* mRNA (Figure 7B) [23]. Therefore, when FBF/PUF activity is lowered in the distal germline (as germ cells leave the mitotic region and enter the transition zone), LIP-1/MKP abundance increases. The result of this extra step of regulation is that MAPK activity is kept low even when FBF levels decrease. Therefore, MAPK inhibition is ensured not only by redundant inhibitors but also by a well-buffered circuitry.

A key unanswered question is whether mammalian MAPK is also subject to homologous redundant controls. Clearly both exist in mammals: PUM2 represses both Erk2 and p38α mRNAs (present work), and MKPs negatively regulate ERK2, p38α and JNK members of the MAPK family [6]. But do they function in the same cells in a redundant fashion? The answer to this question will require removal of both PUF and MKP proteins in vertebrate cells, which remains a challenge for the future.

### PUF Repression of MAPK Signaling in Stem Cells and Cancer Cells

In the C. elegans germline*,* FBF is required for stem cell maintenance [15], and MPK-1 promotes differentiation (either oocyte maturation or apoptosis) [18,19]. Although a vertebrate role for PUF proteins in stem cell maintenance remains a matter of speculation [14,17], recent evidence has given this idea credence. Thus, PUM2 is enriched in germline stem cells and embryonic stem cells [28], and murine PUM2 mutant testes are smaller than normal and contain some agametic seminiferous tubules [32]. Therefore, the role of PUF proteins in stem cell maintenance may be conserved.

The roles of MAPK and MKP in vertebrates are reminiscent of those of MPK-1 and LIP-1 in the C. elegans germline. Vertebrate ERK2 and p38α MAPKs can antagonize stem cell self-renewal and promote differentiation [9,33–35]. In cancer cells, ERK2 and p38α MAPKs are thought to promote apoptosis [36,37]. Indeed, MKPs are often upregulated in human cancer cells, and the MKP inhibition of MAPK activity has been suggested to be critical for human cancer progression [38,39]. Therefore, MAPKs and MKPs affect both continued self-renewal and tumor progression.

In this work, we show that PUF RNA-binding proteins repress MAPK-encoding mRNAs in both C. elegans and hESCs. Indeed, to our knowledge, ERK2 and p38α mRNAs are the first PUM2 targets reported to date. The biological significance of this finding is not known. One simple idea is that PUF represses MAPK expression as part of a larger regulatory circuit designed to maintain stem cells in a naïve state. However, a more complete understanding will require learning the extent of PUM2 repression, the extent of MKP inhibition, and the biological readout of different levels of MAPK activity—all in the same cells. Although this more in-depth understanding remains a challenge for the future, we emphasize here that the PUF and MKP controls of MAPK signaling are broadly conserved and likely work together broadly to control stem cells and tumor progression.

## Materials and Methods

### Nematode strains.

All strains were maintained at 20 °C as described [40], unless noted otherwise. We used the wild-type Bristol strain N2 as well as the following mutants: *LGI*: *gld-1(q485)* [41], *gld-2(q497)* [42,43]; *LGII*: *fbf-1(ok91)* [15], *fbf-2(q738)* [13], *gld-3(q730)* [44], *nos-3(q650)* [45]; *LGIII*: *glp-1(q224)* [46], *mpk-1(ga117)* [2], *mpk-1(ga111)* [24]; and *LGIV*: *lip-1(zh15)* [22].

### In situ mRNA hybridization.

In situ hybridization was carried out using the protocol described [47], with minor modifications. Dissected adult hermaphrodite gonads were fixed (3% formaldehyde, 0.25% glutaraldehyde, 100 mM K_2_HPO_4_ [pH 7.2]) for 3 h at room temperature. After washing three times with PBT solution (1× PBS containing 0.1% Tween 20), gonads were treated with proteinase K (50 μg/ml) for 30 min at room temperature and then refixed in the same solution for 15 min at room temperature. DNA probes were synthesized with digoxigenin-11-dUTP by repeated primer extension. Fixed gonads were incubated for 24 h at 48 °C in a solution containing the DNA probe plus 5× SSC, 50% deionized formamide, 100 μg/ml herring sperm DNA, 50 μg/ml heparin, and 0.1% Tween 20. To visualize the probes, gonads were incubated with alkaline phosphatase−conjugated antidigoxigenin antibody (Roche, 1:2,000 dilution in PBT containing 0.1% BSA) at 4 °C for overnight. After washing several times in PBT (+ 0.1% BSA), staining was developed for 1 h in a solution (100 mM Tris Cl [pH 9.5], 100 mM NaCl, 5 mM MgCl_2_, 0.1% Tween 20, 1 mM Levamosole) containing 4-nitro blue tetrazolium chloride (0.23 mg/ml) and 5-bromo-4-chloro-3-indolyl-phosphatase (0.18 mg/ml) and then terminated in PBT containing 20 mM EDTA.

### Antibody staining.

Dissected gonads were fixed with 3% formaldehyde, 100 mM K_2_HPO_4_ (pH 7.2) for 1 h, and postfixed with cold (−20 °C) 100% methanol for 5 min. Antibody incubations and washes were performed as described [47]. Polyclonal rabbit α-MAPK/ERK antibody (Sc94; Santa Cruz Biotechnology) was used at 1:400 dilution, and monoclonal mouse α-DP-MAPK antibody (Sigma) was used at 1:200 dilution. DAPI staining followed standard methods.

### Western blots.

Blots were prepared by standard procedures. Protein samples were separated on 4%–20% gradient gels (Cambrex), and the blot was probed with polyclonal rabbit α-MAPK/ERK antibody (Sc94; Santa Cruz Biotechnology), α-GFP antibody (Molecular Probes), monoclonal mouse α-tubulin antibody (Sigma), α-actin antibody (MP Biomedicals), and α-FLAG antibody (Sigma).

### Yeast three-hybrid and gel shift assays.

Three-hybrid assays were performed as described [48]. For β-galactosidase assays, cells were grown in selective media to an OD_600_ of 1.0 and mixed with an equal volume of β-Glo (Promega) reagent. Luminescence was measured after 1 h. Gel shift assays were performed as described [49].

### SYTO 12 staining.

SYTO 12 (Molecular Probes) dye was used to estimate the relative numbers of germ cell corpses [19,50]. Animals were incubated in a 33 μM aqueous solution of SYTO 12 for 2 h at 20 °C and then transferred to seeded plates to purge stained bacteria from the intestine. After 30 min, animals were mounted on agarose pads and observed under a fluorescence microscope equipped with Nomarski optics to score SYTO 12−positive germ cells.

### Construction of the *pie-1* promoter::GFP::*fbf-1* ORF+3′UTR transgene.

ORF and 3′ sequences from the *fbf-1* genomic locus (from ATG to 317 bp downstream of the STOP codon) were PCR amplified with flanking attB1 and attB2 sequences and cloned into pDONR201 (Invitrogen) to create pCM3.06, which was sequence verified. A Gateway LR recombination reaction (Invitrogen) was performed between pCM3.06 (entry) and pCM2.03 (destination). pCM2.03 is a bombardment-ready vector containing the *unc-119* rescuing fragment (used for transformant selection [51]), the *pie-1* enhancer and promoter (to drive expression in the germline [52]), GFP with three synthetic introns (from pPD103.87, A. Fire, personal communication), and the attR1::Gateway Cassette B::attR2. The resulting plasmid pCM4.06 contains *unc-119; pie-1* (enhancer + promoter)::GFP::attB1::*fbf-1*ORF+3′UTR::attB2. pCM4.06 was transformed into *unc-119(ed3)* worms by microparticle bombardment [51] to create line JH2012 (genotype: *unc-119(ed3); axIs1459 [CM4.06]*).

### RNA immunoprecipitation and RT-PCR.

Age-synchronous adult Ppie-1::GFP-FBF-1 (JK4091) and Ppie-1::GFP-TUB (AZ224) transgenic animals were grown for 24 h after the L4 stage on NGM plates supplemented with concentrated OP50. Worms were harvested by rinsing plates with M9 buffer, and worms were washed with M9 buffer until the supernatant was clear. Worms were then washed twice with buffer A (20 mM Tris [pH 8.0], 150 mM NaCl, 10 mM EDTA [pH 8.0], 1.5 mM DTT, 0.1% NP-40, 0.02 mg/ml heparin), and worm pellets were frozen at −20 °C. Roughly 0.5 ml of worm pellets was used for affinity purification. Worm lysate was generated by grinding worms with a mortar and pestle under liquid nitrogen in lysis buffer (buffer A plus 1× Complete Protease Inhibitor Cocktail [Roche], 20 U/ml DNase I [Ambion], 100 U/ml RNase OUT [Invitrogen], 0.2 mg/ml heparin), followed by 30 passes with a glass dounce. The extract was then centrifuged twice at 10,000 *g* for 10 min at 4 °C to remove insoluble debris and fat. The protein concentrations of cleared extracts were determined by Bradford assay, and extracts were diluted to 10 mg/ml with lysis buffer. To limit nonspecific interactions with the affinity column, extracts were next precleared by incubation with 15 μl of Immobilized Protein A (Pierce) for 1 h at 4 °C. Then 1.6 μg of mouse α-GFP monoclonal antibody 3E6 (Q•Biogene) prebound to 16 μl of Immobilized Protein A (Pierce) was added to each precleared extract, and the resultant slurries were incubated at 4 °C for 2 h. Beads were then washed once with lysis buffer for 15 min at 4 °C and four times with wash buffer (20 mM Tris [pH 8.0], 150 mM NaCl, 1 mM EDTA [pH 8.0], 10% glycerol, 0.01% NP-40, 1 mM DTT, 10 U/ml RNase OUT) for 15 min at 4 °C. For protein analysis, beads were boiled in Laemmli buffer. To purify RNA, beads were treated with TRIzol reagent (Invitrogen) followed by RNeasy Mini Kit (Qiagen) to purify RNA, following manufacturer's instructions. Reverse transcription reactions were performed using 20 ng of Input or IP RNA in 10-μl reactions with an oligo(dT) primer and SuperScript III reverse transcriptase (Invitrogen). PCR was carried out on the cDNA template for *eft-3, gld-1*, and *mpk-1* in the linear range (33 cycles) using gene-specific primers such that one primer spanned an exon-exon junction. PCR products were resolved on 1% agarose gel and stained with ethidium bromide.

### Human ES cell culture and transfection.

H9 human ES cells with a stably transfected EBNA protein (H9-EBNA) that enables episomal replication of exogenous plasmid containing an Orip site were maintained in defined medium-TeSR medium [53,54] containing 50 ng/ml G418. For the transfection, H9-EBNA cells were dissociated by Dispase (Invitrogen) and seeded onto Matrigel-coated six-well plates. At 24 h after seeding, H9-EBNA cells were transfected with 1 μg of indicated reporter plasmids together with an equal amount of indicated effector plasmids by using Fugene6 reagents (Roche). At 24 h after transfection, the cells were photographed and lysed by RIPA buffer for the Western blot analysis.

## Abbreviations

BGH: bovine growth hormone
DP: dual phosphorylation
EGFP: enhanced green fluorescent protein
FBE: FBF binding element
FBF: *fem-3* binding factor
hESC: human embryonic stem cell
IP: immunoprecipitate
MAP: mitogen-activated protein
MAPK: MAP kinase
mESC: mouse embryonic stem cell
MKP: MAPK phosphatase
NRE: nanos response element
RNAi: RNA interference
3′UTR: 3′ untranslated region
